# QSAR, homology modeling, and docking simulation on SARS-CoV-2 and pseudomonas aeruginosa inhibitors, ADMET, and molecular dynamic simulations to find a possible oral lead candidate

**DOI:** 10.1186/s43141-022-00362-z

**Published:** 2022-06-17

**Authors:** Emmanuel Israel Edache, Adamu Uzairu, Paul Andrew Mamza, Gideon Adamu Shallangwa

**Affiliations:** 1 Department of Pure and Applied Chemistry, Faculty of Science, University of Maiduguri, P.M.B, Maiduguri, Borno State 1069 Nigeria; 2 Department of Chemistry, Faculty of Physical Sciences, Ahmadu Bello University, P.M.B. 1044, Zaria, Kaduna State Nigeria

**Keywords:** QSAR, Homology modeling, Molecular docking, ADMET, MD simulations, SARS-CoV-2, *Pseudomonas aeruginosa*, and iminoguanidine derivatives

## Abstract

**Background:**

In seek of potent and non-toxic iminoguanidine derivatives formerly assessed as active Pseudomonas aeruginosa inhibitors, a combined mathematical approach of quantitative structure-activity relationship (QSAR), homology modeling, docking simulation, ADMET, and molecular dynamics simulations were executed on iminoguanidine derivatives.

**Results:**

The QSAR method was employed to statistically analyze the structure-activity relationships (SAR) and had conceded good statistical significance for eminent predictive model; (GA-MLR: Q^2^_LOO_ = 0.8027; *R*^2^ = 0.8735; *R*^2^_ext_ = 0.7536). Thorough scrutiny of the predictive models disclosed that the Centered Broto-Moreau autocorrelation - lag 1/weighted by I-state and 3D topological distance-based autocorrelation—lag 9/weighted by I-state oversee the biological activity and rendered much useful information to realize the properties required to develop new potent *Pseudomonas aeruginosa* inhibitors. The next mathematical model work accomplished here emphasizes finding a potential drug that could aid in curing *Pseudomonas aeruginosa* and SARS-CoV-2 as the drug targets *Pseudomonas aeruginosa*. This involves homology modeling of RNA polymerase-binding transcription factor DksA and COVID-19 main protease receptors, docking simulations, and pharmacokinetic screening studies of hits compounds against the receptor to identify potential inhibitors that can serve to regulate the modeled enzymes. The modeled protein exhibits the most favorable regions more than 90% with a minimum disallowed region less than 5% and is simulated under a hydrophilic environment. The docking simulations of all the series to the binding pocket of the built protein model were done to demonstrate their binding style and to recognize critical interacting residues inside the binding site. Their binding constancy for the modeled receptors has been assessed through RMSD, RMSF, and SASA analysis from 1-ns molecular dynamics simulations (MDS) run.

**Conclusion:**

Our acknowledged drugs could be a proficient cure for SARS-CoV-2 and *Pseudomonas aeruginosa* drug discovery, having said that extra testing (in vitro and in vivo) is essential to explain their latent as novel drugs and manner of action.

**Supplementary Information:**

The online version contains supplementary material available at 10.1186/s43141-022-00362-z.

## Background

Coronaviruses are separated into four kinds: Alphacoronavirus, Betacoronavirus, Gammacoronavirus, and Deltacoronavirus [1]. Many species, including humans, have been shown to suffer respiratory, intestinal, neurological disorders, and hepatic caused by these viruses, particularly Betacoronavirus [2]. The World Health Organization (WHO) named it 2019-novel coronavirus (2019-nCoV) after determining the involvement of coronavirus in COVID-19 [3] (https://www.who.int/emergencies/diseases/novel-coronavirus-2019). Referable to world health emergencies, the International Committee of Coronavirus Study Group (ICCSG) proposed using the named severe acute respiratory syndrome coronavirus 2 (SARS-CoV-2) for 2019-nCoV [4]. Because of the onset of pandemic crises around the world, SARS-CoV-2 has now developed a major community health anxiety [5]. The WHO has labeled COVID-19 a community health matter of global concern because of its speedy spreading and ever-increasing procreation/transmission number [6]. As of August 13, 2021, the number of confirmed cases is 205,338,159 and the number of confirmed deaths is 4,333,094 (https://www.who.int/emergenciess/diseases/novel-coronavirus-2019). During infection with SARS-CoV-2, the amount of *Pseudomonas aeruginosa* increases, encouraging inflammation by accelerating the recruitment of inflammatory cells and increasing the level of angiopoietin II (https://www.who.int/emergenciess/diseases/novel-coronavirus-2019). The protease is one of the numerous products of the SARS-CoV-2 binding target [7, 8]. Drugs remain the only therapeutic option for *Pseudomonas aeruginosa* and SARS-CoV-2, despite efforts to create a vaccine [9]. Due to different medication resistance scenarios around the world, the number of people dying annually from *Pseudomonas aeruginosa* and SARS-CoV-2 is steadily rising [9, 10]. Given the lack of viable medicines and the continual growth in transmission numbers and fatality cases. Computer-aided drug discovery (CADD) [11] could be a good strategy to discover hit drugs for *Pseudomonas aeruginosa* and SARS-CoV-2 treatment. This computer-aided drug design and development technique will cut down on the cost and time it takes to find new therapeutic candidates [12]. Ahmad et al. have reported the docking, molecular dynamic simulation, and MM-PBSA studies of *Nigella Sativa* compounds to find likely normal antiviral drugs for SARS-CoV-2 treatment [13]. Amin and his coworkers have reported the use of Monte Carlo-based QSAR, virtual screening, and molecular docking study of some inhouse molecules as inhibitors of COVID-19 [14]. Several CADD methods have been used to study and design hit drugs such as anticancer [15, 16], monoamine oxidase B inhibitors [17], antimicrobial [18], dengue virus [19], and antidiabetic [20] drugs, etc. To select a chemical compound as a viable treatment, the following in silico technique such as quantitative structure-activity relationship (QSAR), molecular docking simulation, absorption, metabolism, excretion, and distribution (ADME), and dynamics modeling of many drugs from known drugs library are used against the target receptors. In the present research, we executed QSAR studies on some chemical libraries using genetic function approximation-multiple linear regression (GFA-MLR). The best model out of the many generated model will be systematically analyzed. The results gained from these methods were equated for validation. Next, we perform the homology modeling of our query protein, then docking simulation to obtain information about the main interaction types from the built model receptor active pocket. Their drug-likeness parameters of the most beneficial docked compound were assessed via in silico approach. Finally, simulations were executed to assess the dynamic stableness of the docked receptors. The current modeling study would offer understanding into the structural demands of these COVID-19 and *Pseudomonas aeruginosa* inhibitors and may aid in planning novel drugs.

## Methods

Density function theory (DFT/B3LYP) with the 6-31G+ (d, p) basis sets in Gaussian 09 were used to thoroughly optimize the geometries of the iminoguanidine derivatives (PubChem database accession number AID_131512). The PaDEL v2.20 program [21] was used to calculate the properties for QSAR analysis. The association between one dependent variable (pMIC_50_) of 25 compounds and various independent variables was studied using GA-MLR statistical techniques. The genetic approximation (GA) technique which is included in QSARINS v2.2.4 [22] was used to perform multiple linear regression (MLR) analysis of the molecular descriptors. By dividing the database into two groups, a training set to construct the quantitative model and a test set to confirm the proficiency of the molded model. All the minimum inhibitory concentration (MIC) activity data in the experiments were first translated to the negative logarithm of MIC (pMIC_50_ = −log10 (MIC)). Table S1 shows the chemical structures of iminoguanidine compounds as well as their activity levels. To test the internal validity of the regression model, we employed the LOO (leave-one-out) approach [23, 24]. This (Q2LOO) is the most frequent way of determining a model’s inner prediction ability. We used randomized validation [25] (Q2rand, R2rand), root mean square error of the training set (RMSEc), and coefficient of determination to assess model robustness in addition to (Q2LOO). For external validation, we used Q2F1 [26], Q2F2 [27], and Q2F3 [28], as well as the concordance correlation coefficient (CCC) and root mean square error of prediction (RMSEp) as recommended by the Organization for Economic Cooperation and Development (OECD) [29]. QL2OO > 0.5, R2 > 0.6, 0.85 ≤ k ≤ 1.15 or 0.6, 0.85 ≤ k’ ≤ 1.15 [30], Q2F1 > 0.5, Q2F2 > 0.5, Q2F3 > 0.5, and CCC > 0.80 are some of the evaluation criteria.

### Homology modeling

To build the initial structure for the molecular docking and MD simulation studies, homology modeling of *Pseudomonas aeruginosa* and SARS-CoV-2 secondary structure was undertaken. The NCBI protein sequence database (http://www.ncbi.nlm.nih.gov) was used to search the sequence of amino acids for *Pseudomonas aeruginosa* and SARS-CoV-2. A BLASTp search against the Brookhaven Protein Data Bank (PDB) was used to select the template structure, which was based on sequence identity. The chain A, SARS-CoV-2 virus main protease (PDB 7BUY) as the query structure from NCBI and the identified template structures (PDB code: 5R7Y, 6XA4, 7BRO, 7CB7, 7CBT, 7CWC, and 7KFI) were modest in BLAST results. According to the BLAST results for RNA polymerase-binding transcription factor DksA (plasmid) [*Pseudomonas aeruginosa*] (query id: QNI 16641.1) and the identified templates PDB 4IJJ (query cover: 95%, *E*-value: 5e−31, percentage identity: 44.03%) and PDB 1TJL (query cover: 85%, *E*-value: 1e−19, percentage identity: 35%) were used. Following that, using ClustalX [31], the coordinates for the query structure were assigned from the template structure using pairwise sequence alignment. MODLOOP Server [32] was used to correct irregular secondary structures. The 3D protein structures were then built using MODELLER 10.1 [33]. As a result, the model with the lowest discrete optimized protein energy (DOPE) score was chosen, and the model was then energy minimized (add hydrogen and Gasteiger charge) using Chimera v1.10.2 software with the AMBER FF14SB force field. SAVES server was used to calculate stereochemical characteristics, the atomic model’s (3D) compatibility with its amino acid residues, bond lengths, bond angles, and side-chain planarity were all utilized to verify the model’s quality. PROCHECK [34] was used to calculate Ramachandran plots to verify the stereochemical quality of modeled protein structures. Verify3D [35] and ERRAT [36] were used to create an environment profile. WHATIF was used to investigate residue packing and atomic contact, whereas WHATCHECK was utilized to calculate the Ramachandran plot’s Z Score [37]. Using PyMOL, the RMSD was calculated by superimposing the 3D modeled protein with the template.

### Structure-based virtual screening and docking

To perform molecular docking simulations and virtual screening, we utilized Autodock Vina [38] with the PyRx [39] interface tool. Before being converted to PDBQT format, all the optimized ligand molecules and the modeled proteins were uploaded into the PyRx work station. Then, using the Lamarckian genetic algorithm, virtual screening was performed with the following parameters: exhaustiveness 8, the grid for SARS-CoV-2 was set to center_x = 14.2355, center_y = 0.4381, center_z = 5.5567, size_x = 38.0396286631, size_y = 65.9951690292, and size_z = 58.8759282303, while the grid for *Pseudomonas aeruginosa* was set to center_x = 47.9912699312, center_y = 38.6282164717, center_z = 30.4668261785, size_x = 96.3490410625, size_y = 84.4136676486, and size_z = 103.798747643. Discovery studio 2020 client was used to sort out the most proficient docked ligand conformations and examine the bond lengths and binding interactions. Azithromycin, Doxycycline, Levofloxacin, Fluoroquinolone, Chloroquine, Ritonavir, Ruxolitinib, and Ampicillin (Table S1) were used as control drugs against SARS-CoV-2 virus main protease and *Pseudomonas aeruginosa* proteins, respectively.

### Molecular dynamics simulations (MDS)

MDS is a thermodynamic-based procedure that aids in the investigation of dynamic changes encountered in protein-ligand complexes. To certify the integrity of the ligand-protein combination in our investigation, we used MDS to examine the best ligands screened in previous phases with their corresponding proteins. The molecular docking complexes were simulated using the NAMD 2.13 Win64-multicore version [40], which included the Chemistry at HARvard Macromolecular Mechanics (CHARMM 36) force field [41] and the TIP3P water model. Several co-time approaches were applied, with a 2fs integration time step. The CHARMM-GUI web service [42] was used to produce ligand topology and parameter files, produce psf files of protein-ligand complexes, water box, and neutralize the system with potassium (K^+^) and chloride (Cl^-^) ions. The simulation/production (NPT) ran for 1 ns with 5000 steps of minimization (NVT). The temperature was kept constant at 303 K using a Langevin thermostat. The system’s perimeter was surrounded by periodic boundary conditions. Visual molecular dynamics (VMD) [43] was utilized for the visualization of the complex.

## Results

In the current study, about 1500 descriptors from PaDeL v2.20 using DFT (B3LYP/6-31G+(d,p)) were computed. Descriptors compete for space in the 25 compounds studied; on these descriptors, a genetic approximation-multiple linear regression (GA-MLR) was employed. As a result, all descriptors with a low correlation coefficient value concerning the dependent variable were first discarded. Also, descriptors with a correlation coefficient larger than 0.95 are eliminated from our data matrix to reduce ambiguity. The GA analysis selects the remaining descriptors, which are then employed in the creation of MLR models. QSARINS software v2.2.4 [44, 45] was used to divide the entire dataset into training and test sets at random. From the training set, the GA-MLR model with the highest coefficients of determination and explained variance in “leave one out” cross-validation prediction, and reasonable ability to predict MIC_50_ values of test set chemicals was chosen. The extended QSAR model is given in the equation below:

**PMIC50 =**  ‐ **7.3643**(**ATSC1s**) **+ 0.0274** (**TDB9s**) ‐ **1.0399 Model 1**

The more important the regression model, the lower the *p*-value (Table 1), and all of the descriptors’ *p*-values were less than 0.05, indicating that they were statistically significant at the 95% level. Edache et al. [46] stipulated that the descriptors developed in a QSAR model should not be inter-correlated with one another. If descriptors are heavily connected among themselves, the model will be highly unstable. As a result, the developed model is statistically insignificant if the VIF is developed to evaluate descriptor inter-correlation. The VIF values of both descriptors in this model are 1.23 which are less than the threshold value of 10 [47]. Table 1 shows the parameters utilized in the final model have relatively low inter-correlation based on VIF analysis. The mean effect (MF) value was calculated for each descriptor to determine its relative importance and contribution to the model. ATSC1c is a molecular descriptor based on Centred Broto-Moreau autocorrelation with lag 1/I-state weighting. The descriptor is related to pMIC50 in a good way. It is assumed that increasing the ATSC1c descriptor by 76% boosts the bioactivity of drugs or anti-*Pseudomonas aeruginosa* activity. The final descriptor is TDB9s, which stands for 3D topological distance-based autocorrelation - lag 9/weighted by I-state. A 24% rise in the value of this descriptor increases the inhibitory activity of a compound.

**Table 1:** Statistical features for GA-MLR models with relevant descriptors

Variable	Coeff.	Description	Std. coeff.	Std. err.	(+/−) Co. int. 95%	*p*-value	VIF	MF
Intercept	−1.0399			0.2891	0.6161	0.0024		
ATSC1c	−7.3643	Centered Broto-Moreau autocorrelation - lag 1/weighted by I-state	−0.6614	1.0755	2.2923	0.0000	1.23	0.760
TDB9s	0.0274	3D topological distance-based autocorrelation - lag 9/weighted by I-state	0.4866	0.0054	0.0116	0.0001	1.23	0.240

Internal and external cross-validation was used to assess the model’s predictive potential. The model’s results, as well as their regression statistics, are presented in Table S2 and S3. Fig. S1 and S2 present the plots of experimental activity versus predicted activity for the training set and the test set compounds, calculated using model 1. Fitting’s criteria, internal validation criteria, and external validation criteria values for the model were judged according to the acceptable threshold [48–50]. Furthermore, the residual for the predicted pMIC50 values for both the training and test sets are plotted against the experimental pMIC50 values in Fig. S3 and S4. The model did not show any proportional or systematic inaccuracy since the propagation of residuals on both sides of zero is random (Fig. S3). The residuals calculated using prediction by leave-one-out (LOO) (Fig. S4) confirm the claim [51]. Each component’s leverage results can be computed and plotted against standardized residuals, allowing for graphical spotting of outliers and influential compounds in a model. The hat matrix (H’s) diagonal elements indicate the molecules’ leverages, which may be computed using the formula below:

1 $$H=X{\left({X}^TX\right)}^{-1}{X}^T$$

where *X* is the training set matrix and *X*^*T*^ denotes the transpose of *X*.

Fig. S5 and S6 show the applicability zone as a squared region defined by a 2.5 bound for residuals and leverage values or warning leverage (*h*^∗^). This *h*^∗^ is the threshold value for *X* computed as a parameter for prediction for a certain model and it is stated as follows:

2 $${h}^{\ast }=3\left(P+1\right)/n$$

where *p* signifies the number of model parameters and *n* constitutes the number of compounds [29]. Fig. S5 shows that the test set’s compound 15, a response outlier and compound 16, a structurally influential outlier is outside of this square area. While in Fig. S6 using prediction by leave-one-out (LOO), compounds 15 and 20 of the training and test set with standardized residuals exceeding 2.5 standard deviation units are response outliers. A structurally influential outlier is compound 16 from the test set, which is not within the cut-off value of h* = 0.5. Surprisingly, one of the training sets compounds and two of the validation compounds both had leveraged greater than the threshold value and low residuals. As previously established by Jaworska and coworker [52] present, compounds with hat matrix (*H*'s) greater than h* alleviate the model and make it predictive for new compounds that differ structurally from the training set [53]. This is only true when the training compound residuals are low. To ensure that all molecules from the estimate set were within the model domain, we used the Insubria graph [54]. The leverages for prediction set vs predicted values are plotted in the graph (Fig. S7). Based on molecular similarity to the training set compounds (leverage value) and the predicted value of pMIC50, we identified the model’s reliable prediction zone with this figure. We discovered that 50% of the molecules in the test set fit into the model’s applicability zone. Compounds 12, 16, and 18 were discovered to be beyond the zone. To ensure model quality, the Y-scrambling process was used to confirm the absence of chance correlations in the initial GFA-MLR model. As projected, Fig. S8-S10 shows a satisfactory model was obtained.

### Homology modeling

**Fig. 1:**
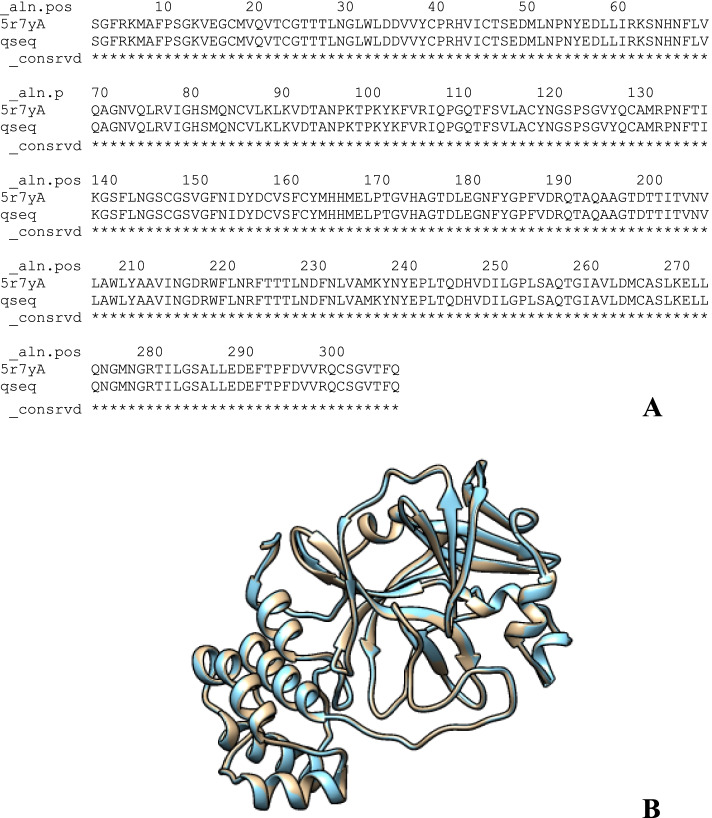
In homology modeling, multiple sequence alignment was used. **A** The crystal structure of COVID-19 main protease in complex with carmofur was named qseq. **B** Predicted structure in general with chain A, 3C-like proteinase (severe acute respiratory syndrome coronavirus 2)

Homology modeling is typically used to create protein models and follows a set of well-defined and widely acknowledged procedures [55]. During the homology modeling phase, we aim for an experimentally determined structure with the COVID-19 virus main protease and RNA polymerase-binding transcription factor DksA (plasmid) that has a high “sequence identity.” Chain A, 3C-like proteinase (severe acute respiratory syndrome coronavirus 2) target and template PDB I.D: 5R7Y protein sequences were aligned as indicated in Fig. 1A. The homology model of COVID-19 primary protease in association with carmofur was built using crystal structures of chain A, 3C-like proteinase (PDB: 5R7Y) as a template, and then modified by loop modeling. Figure 1B shows an overview of the aligned template and target sequence’s projected 3D structure with the alignment calculated using PyMOL molecular viewer yielded an RMSD value of 0.169.

**Fig. 2:**
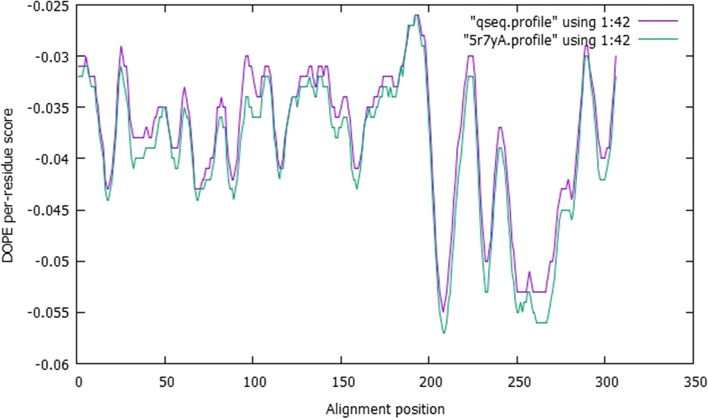
The model profile was superimposed over the template profile

In this investigation, the Discrete Optimized Protein Energy (DOPE) score [56], which is included in the MODELLER package and is extensively used to assess the quality of 3D models. The DOPE score values for the SARS-CoV-2 models are presented in Table 2. Models with a lower DOPE score and high molpdf values were regarded as structurally sound and reliable in terms of energy values. The model with a DOPE score of −36285.0 and a molpdf value of 1550.75635 (model 1) was chosen in the case of the COVID-19 virus. The model and templates were superimposed according to the DOPE score profiles as presented in Fig. 2. The long active site loop between residues 10–50, 100–120, and 280–310, as well as the long helices at the C-terminal and N-terminal ends of the target sequence, has relatively high energy, according to the plotted DOPE score profile. This lengthy loop interaction with the region makes up the active sites.

**Table 2:** The models and DOPE score generated for severe acute respiratory syndrome coronavirus 2

Filename	molpdf	DOPE score	GA341 score
qseq.B99990001.pdb	1550.75635	−36285.00000	1.00000
qseq.B99990002.pdb	1594.63806	−36061.37891	1.00000
qseq.B99990003.pdb	1698.69629	−36243.19531	1.00000
qseq.B99990004.pdb	1557.67419	−36204.93359	1.00000
qseq.B99990005.pdb	1516.13452	−36275.48828	1.00000

**Fig. 3:**
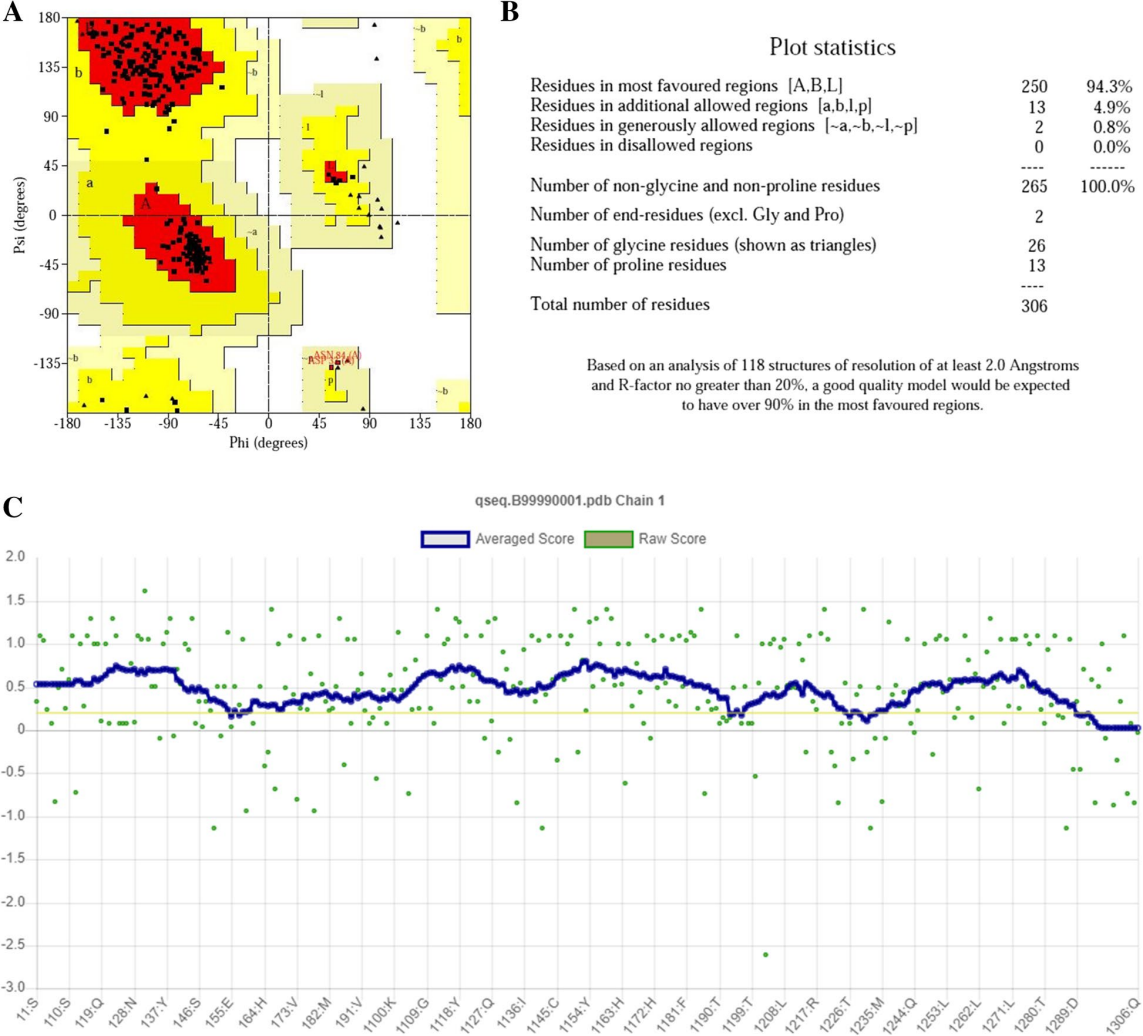
Validation of structure using **A** Ramachandran plot, **B** Ramachandran plot statistics of the homology modeled SARS-CoV-2 virus main protease, and **C** Verify3D for structure validation

Different techniques, such as PROCHECK (Ramachandran plot), PROVE, ERRAT2, and VERIFY 3D, were used to assess the 3D model’s structural integrity. The modeled protein’s Ramachandran plot (Fig. 3A, B) shows that 93.3% (250 aa) of the total residues are in the most favored regions and 4.9% (13 aa) are in additional allowed regions, and 0.8% (2 aa) are in the generously allowed regions, indicating a high-quality model. The modeled protein’s Verify3D plot (Fig. 3C) was obtained, and it showed PASS. The ERRAT2 overall quality factor for the COVID-19 model is around 88.26% (Fig. S11A).

**Fig. 4:**
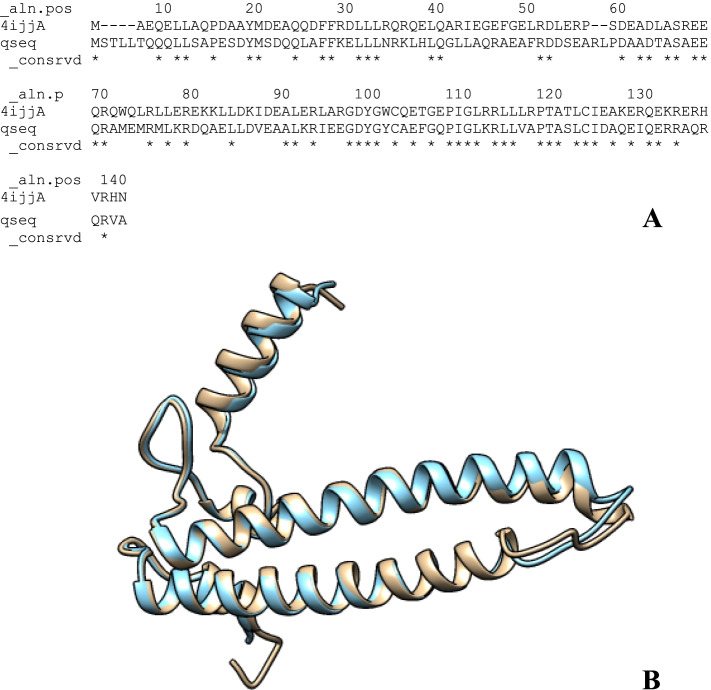
Alignment of RNA polymerase-binding transcription factor DksA (plasmid) amino acid sequences with the crystal structure of transcription factor DksA2 from Pseudomonas aeruginosa (PDB: 4IJJ)

**Fig. 5:**
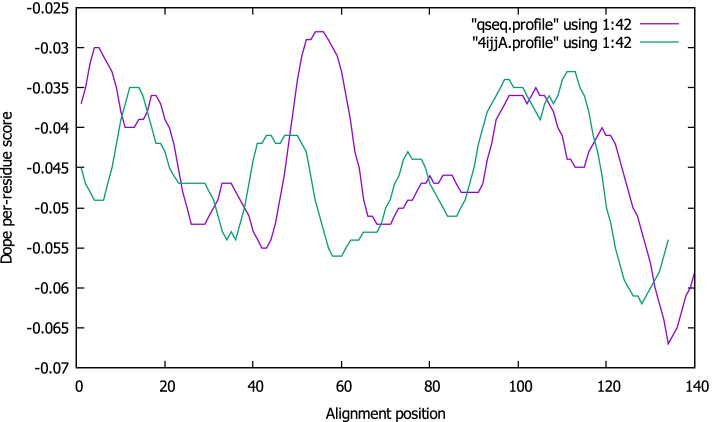
DOPE score profiles for the query sequence (qseq) and templates of PDB: 4IJJ

**Fig. 6:**
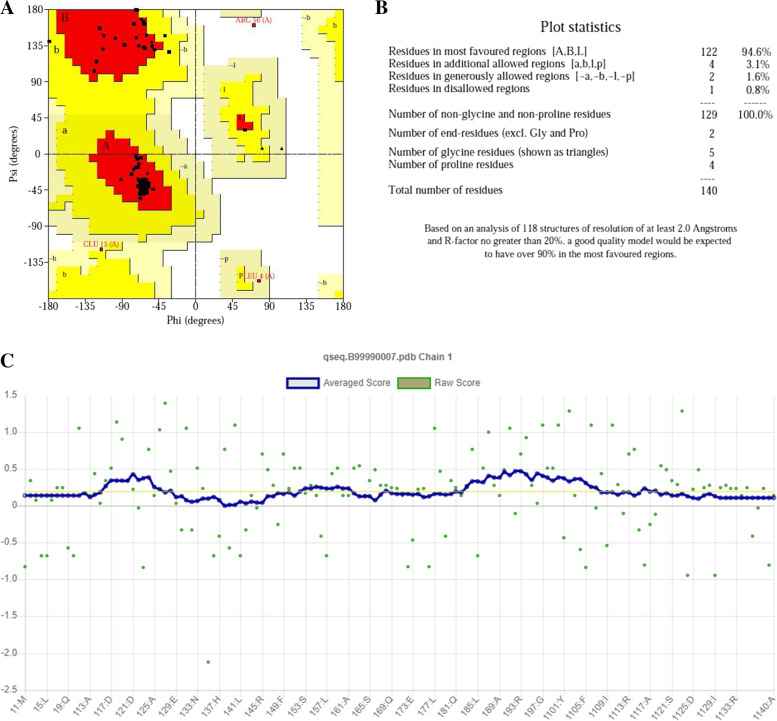
**A** Ramachandran plot, **B** Ramachandran plot statistics of the homology modeled RNA polymerase-binding transcription factor DksA (plasmid), and **C** Verify3D for structure validation

The overlapping of the structure of transcription factor DksA2 from *Pseudomonas aeruginosa* and RNA polymerase-binding transcription factor DksA models shows great similarity, possibly due to the homology modeling procedure (Fig. 4A). Ten (10) PDB structures were generated, using MODELLER 10.1, and the best receptor model was chosen based on the DOPE assessment method as presented in Table 3. Figure 4 shows an overview of the aligned template and target sequence’s projected 3D structure with the alignment calculated using PyMOL yielded an RMS value of 0.288. The model and templates were superimposed according to the DOPE score profiles as shown in Fig. 5. To evaluate the reliability of RNA polymerase-binding transcription factor DksA models built for docking purposes, we used a Ramachandran plot. These methods identify the Psi/Phi angle distribution in the 3D model within the allowed or disallowed regions. Ramachandran plot (Fig. 6) of the modeled protein represents 94.6% (122 aa) of the total residues in the most favored regions, 3.1% (4 aa) in additionally allowed regions, residues in generously allowed regions is 1.6% (2 aa), and 0.8% (1 aa) residues in disallowed regions, indicating a good quality model. The modeled protein’s Verify 3D plot (Fig. 6C) was obtained, and it showed PASS. The ERRAT2 overall quality factor for the RNA polymerase-binding transcription factor DksA model is around 91.667% (Fig. S11B).

**Table 3:** The summary of successfully predicted models for *Pseudomonas aeruginosa*

Filename	molpdf	DOPE score	GA341 score
qseq.B99990001.pdb	530.59882	−12190.35352	1.00000
qseq.B99990002.pdb	676.84918	−11866.63574	1.00000
qseq.B99990003.pdb	560.30042	−12064.89746	1.00000
qseq.B99990004.pdb	554.58264	−12033.17285	1.00000
qseq.B99990005.pdb	574.89307	−12037.96191	1.00000
qseq.B99990006.pdb	767.39117	−11957.61328	1.00000
qseq.B99990007.pdb	506.20578	−12191.88086	1.00000
qseq.B99990008.pdb	576.82599	−12180.59766	1.00000
qseq.B99990009.pdb	561.56323	−11841.31934	1.00000
qseq.B99990010.pdb	559.25763	−12143.47461	1.00000

### Molecular docking simulations

**Fig. 7:**
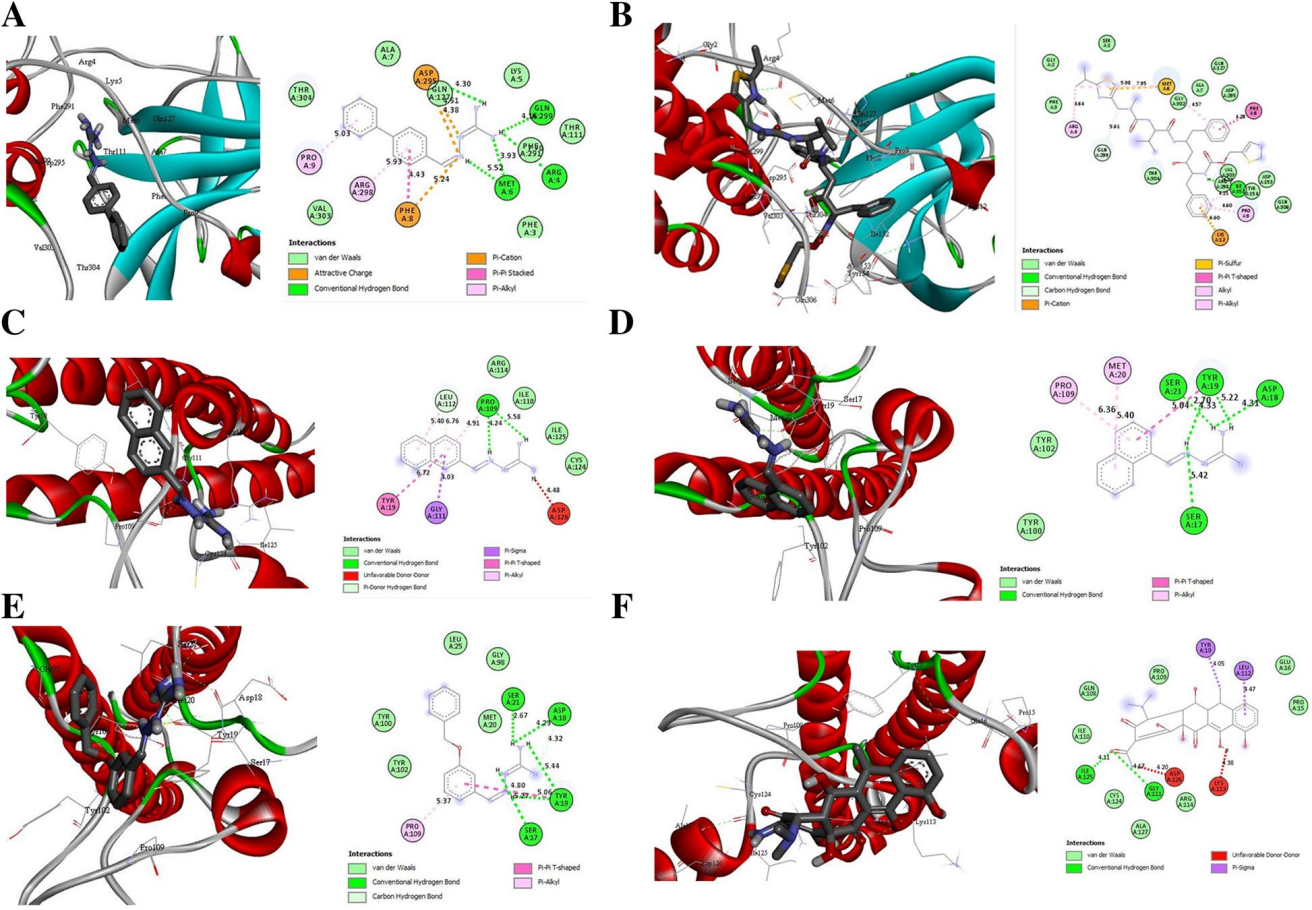
Docking interaction of COVID-19 virus main protease and RNA polymerase-binding transcription factor DksA with ligands. **A** Compound 18, **B** Ritonavir, **C** compound 7, **D** compound 12, **E** compound 15, and **F** Doxycycline. The 2D diagram shows the interactions of the amino acid residues in the binding pores. Colors of residues indicate the types of interactions and bond distances (Å) are shown on each interaction (color figure online)

The selected configurations from the docking result are required in molecular docking simulation to determine the theoretical correctness of the produced complex structure between ligand and receptor. The active site of the modeled SARS-CoV-2 proteinase and modeled RNA polymerase-binding transcription factor DksA was docked by all 25 studied compounds and 8 controls or tested drugs. Within the defined active site, the docking program generates several poses with varied placements. The binding affinity score was used to determine the final ranking of the ligand docking postures. The binding affinity score of all the studied compounds and the control drugs are presented in Table S4. The binding poses of the best ligand and standards with the lowest binding affinity are depicted in 3D and 2D diagrams in Fig. 7. The ligand number 18 has the highest binding affinity against SARS-CoV-2 virus main protease, at −8.7 kcal/mol, followed by the control (Ritonavir) at −8.4 kcal/mol. As illustrated in Fig. 7A, compound 18 with the highest binding affinity formed hydrogen bond interactions with Asp 295 (4.30 Å), Gln299 (4.15 Å), Arg4 (7.70 Å), Met6 (3.93 Å), and (5.52 Å), respectively. It also forms hydrophobic contacts with Pro9 (5.03 Å), Arg298 (5.93 Å), and Phe8 (4.43 Å), as well as electrostatic interactions with Phe8 (5.24 Å), Asp295 (4.51 Å), and (4.32 Å). Ritonavir formed various types of interactions between amino acids and various groups of atoms attached to the control. Ile152 (4.73 Å) formed conventional hydrogen bond interactions with the -NH group, Gln299 (5.61 Å) formed carbon-hydrogen bond interactions with the -CH_2_N- group, and Lys12 (4.73 Å) formed carbon-hydrogen bond interactions with the -CH_2_N- group as illustrated in Fig. 7B, a pi-donor hydrogen bond interaction with the terminal benzene ring was also created. Against modeled RNA polymerase-binding transcription factor DksA model protein, Doxycycline showed better binding affinity than ligand numbers 7, 12, and 15 (Table S4). Doxycycline has the maximum negative binding affinity of −7.2 kcal/mol, followed by Ritonavir with −6.7 kcal/mol. Compounds 7, 12, and 15 have a better binding affinity (−6.5 kcal/mol) than the rest of the studied compounds. From (Fig. 7C–E), compound 7 forms two conventional hydrogen bond interactions with the active site residues Pro109 (4.24 Å) and (5.58 Å), it also forms one unfavorable donor-donor interaction with Asp126 (Fig. 7C). Compound 12 forms five conventional hydrogen bonds and two hydrophobic interactions as presented in Fig. 7D. While compound 15 (Fig. 7E) also have 5 conventional hydrogen bonds with Ser21 (2.67 Å), Asp18 (4.23 Å), Tyr19 (5.06 Å), Ser17 (5.27 Å), and Tyr19 (5.44 Å). A carbon-hydrogen bond with Asp18 (4.32 Å) and two hydrophobic interactions with Pro109 (5.37 Å) and Tyr19 (4.8 Å), respectively. Lastly, the control drugs (Doxycycline) have two conventional hydrogen bonds with Ile125 (4.11 Å) and Gly111 (4.17 Å) and two unfavorable donor-donor interactions with Asp126 and Lys113. The unfavorable interactions found in compound 7 and Doxycycline disqualified them for further analysis. Compound 15 (Fig. 7E) has more hydrogen bonds than compound 12; hence, compound 15 was used for molecular dynamics simulations.

**Fig. 8:**
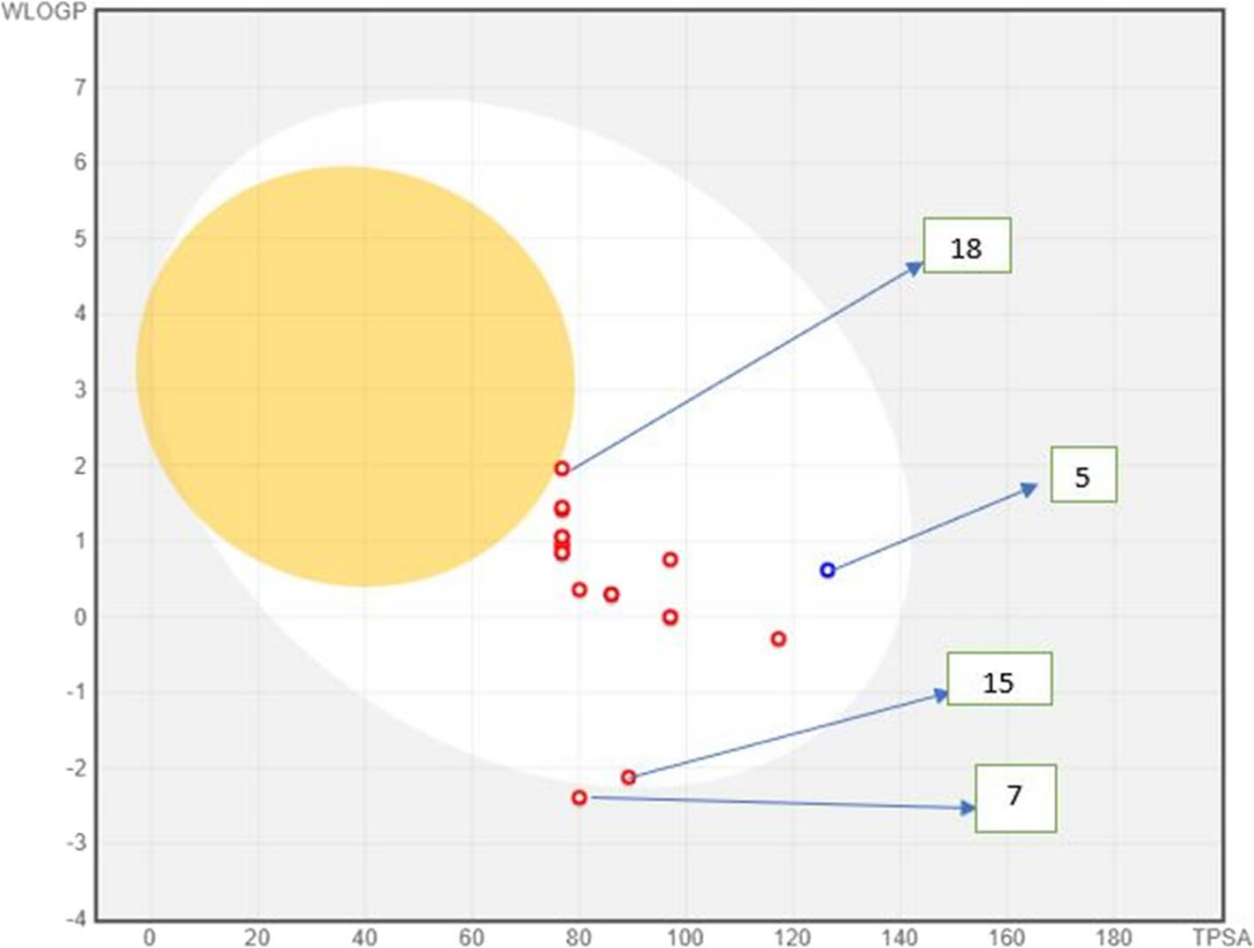
The Egan egg method was used to evaluate the ligands that were studied (http://www.swissadme.ch/)

SwissADME (http://www.swissadme.ch/) was employed to estimate the drug-likeness of our inhibitors, including their ADME inside the body [57]. The SwissADME program’s Egan BOILED-Egg method was utilized to determine the inhibitors’ absorption in the intestinal system and the brain. The BOILED-Egg (Brain Or IntestinaL EstimateD permeation predictive model), also known as the Egan egg, provides a threshold (WLOGP ≤ 5.88 and TPSA ≤ 131.6) as well as a well-defined graphic illustration of how far a chemical structure deviates from the ideal for optimal absorption [58]. In Fig. 8, the molecules in the white part of this 2D graphical representation are predicted to be quietly absorbed by the gastrointestinal (GI) tract, whereas the yolk area represents chemicals that can passively cross the blood-brain barrier (BBB). None of the chemicals are absorbed by the brain, as seen in the graph. The gastrointestinal absorption of all inhibitors was within tolerable limits (WLOGP ≤ 5.88 and TPSA ≤ 131.6) (Fig. 8). The blue dots (compound 5) indicate molecules that P-glycoprotein is predicted to effluate from the central nervous system (CNS), whereas the remaining compounds (red dots) indicate compounds that P-glycoprotein is predicted not to effluate from the CNS.

**Fig. 9:**
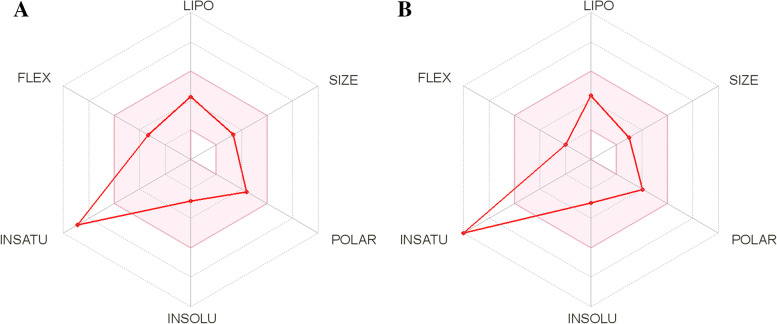
The bioavailability radar of **A** compound 15 **B** compound 18 (http://www.swissadme.ch/)

Figure 9 depicts the bioavailability radar of the compounds for six physicochemical characteristics. The bioavailability radars of compounds 15 (Fig. 9A) and 18 (Fig. 9B) demonstrated a quick assessment of drug-likeness. The bioavailability radar takes into account the following six physicochemical characteristics: (1) lipophilicity (XLOGP3 between 0.7 and +5.0), (2) size (molecular weight between 150 and 500 g/mol), (3) polarity (total polar surface area between 20 and 1302), (4) solubility (log S less than 6), (5) saturation (fraction Csp3 less than 0.25), and (6) flexibility (the number of rotatable bonds not more than 9). The pink area reflects the optimal range of these traits [59], while the red line shows each compound’s properties. In Fig. 9, the in-saturation of both compounds is visible, whereas the other characteristics are inside the pink area. As a result, we can conclude that these chemicals are expected to be bioavailable when taken orally.

### The MD simulations of the docked complexes

**Fig. 10:**
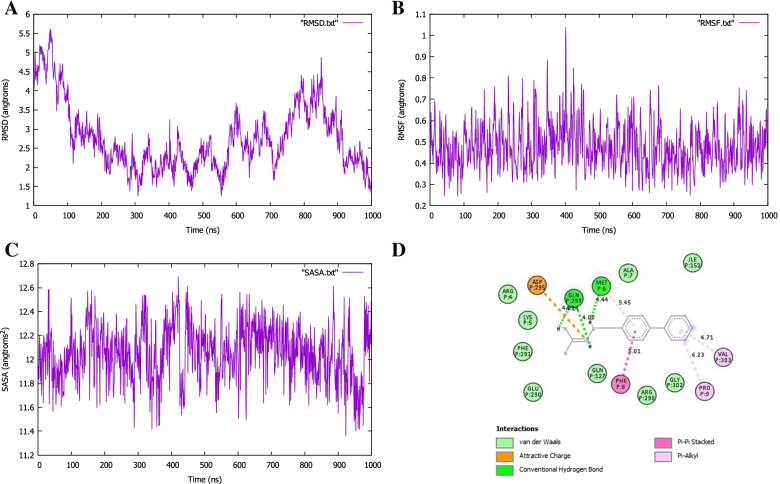
MD simulation study of COVID-19 main protease in complex with carmofur

**Fig. 11:**
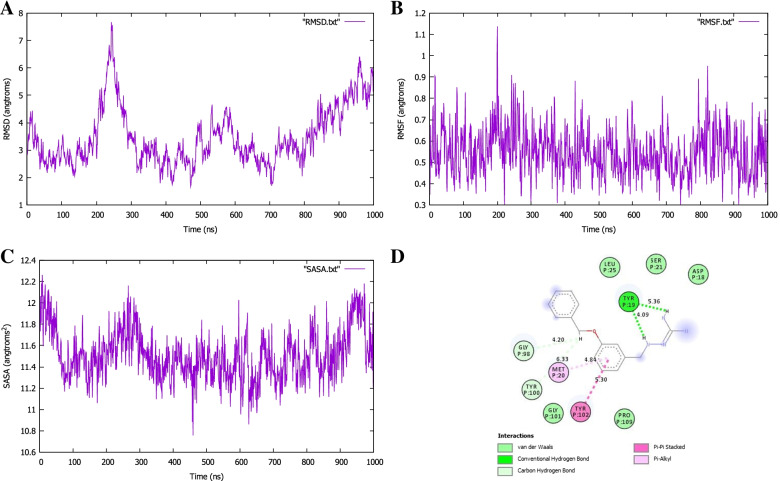
MD simulation study of the RNA polymerase-binding transcription factor DksA (plasmid)

The MDS was executed to assess the constancy of the docked complexes. The complex stability was investigated by calculating the backbone using root-mean-square deviation (RMSD), root means square fluctuation (RMSF), and solvent accessible surface area (SASA). The RMSD of the Cα atoms in the docked complexes was assessed to see the structural deviations all over the simulation trajectory. The complexes reach their stable state after 1-ns which showed structural stability. The RMSD value of the SARS-CoV-2 protein complex is 2.76 Å and that of the *Pseudomonas aeruginosa* protein complex is 3.47 Å. As shown in Fig. 10A, the fluctuation of the SARS-CoV-2 protein complex was within acceptable range with RMSD less than 3 Å indicating the stability of the protein complex conformation. The fluctuation of the *Pseudomonas aeruginosa* protein complex (Fig. 11A) exhibited an increasingly RMSD value toward the end of the simulation. To examine the local differences of protein flexibility, the RMSF results were calculated by taking the average of all backbone residues of atoms (Figs. 10 and 11B). The changes shown below play a significant role in protein complex flexibility, influencing protein-ligand activity and stability. The high RMSF value demonstrates more flexibility, with a maximum level of fluctuation in the residue positions of 400 ps at 1 (Fig. 10B) and 200 ps at 1.1 (Fig. 11B), but the low RMSF value exhibits extremely limited movements. The solvent-accessible surface area of the simulated complexes was also analyzed. These simulation descriptors correlate with the surface volume of the complexes where a higher SASA profile indicates the expansion in the surface area. The SASA trend in the simulated complexes was higher, indicating an increase in surface volume. These simulated complexes, on the other hand, did not show a high level of SASA deviations, indicating that no major modifications to the protein’s surface area were occurring. The SASA for both complexes was calculated using surface racer v5 [60]. The SASA for the SARS-Cov-2 protein complex (Fig. 10C) has a total accessible surface area of 16548.26 Å^2^, polar accessible area of 9668.47 Å^2^, and non-polar accessible surface area of 6879.79 Å^2^, while the *Pseudomonas aeruginosa* protein complex (11C) has a total accessible surface area of 11688.04 Å^2^, polar accessible area of 6411.80 Å^2^, and non-polar accessible surface area of 5276.24Å^2^ (Table 4).

**Table 4:** The SASA results for the SARS-CoV-2 and *Pseudomonas aeruginosa* protein complex

The surface area of SARS-CoV-2	The surface area of *Pseudomonas aeruginosa*
Number of non-HOH, non-H atoms = 2703	Number of non-HOH, non-H atoms = 1213
Probe radius = 1.40	Probe radius=1.40
TOTAL ASA = 16548.26	TOTAL ASA = 11688.04
TOTAL MSA = 0.00	TOTAL MSA = 0.00
Polar ASA = 9668.47	Polar ASA = 6411.80
Non-polar ASA = 6879.79	Non-polar ASA = 5276.24
Polar MSA = 0.00	Polar MSA = 0.00
Non-polar MSA = 0.00	Non-polar MSA = 0.00
Total backbone ASA = 3209.50	Total backbone ASA = 1390.08
Total backbone MSA = 0.00	Total backbone MSA = 0.00
Polar backbone ASA = 2089.27	Polar backbone ASA = 859.84
Non-polar backbone ASA = 1120.23	Non-polar backbone ASA = 530.23
Polar backbone MSA = 0.00	Polar backbone MSA = 0.00
Non-polar backbone MSA = 0.00	Non-polar backbone MSA = 0.00
Polar side chain ASA = 7579.21	Polar side chain ASA = 5551.96
Non-polar side chain ASA = 5759.55	Non-polar side chain ASA = 4746.01
Polar side chain MSA = 0.00	Polar side chain MSA = 0.00
Non-polar side chain MSA = 0.00	Non-polar side chain MSA = 0.00
+charge ASA = 1234.27 −charge ASA = 1092.32	+charge ASA = 1252.44 −charge ASA = 1540.53
+charge MSA = 0.00 −charge MSA = 0.00	+charge MSA = 0.00 −charge MSA = 0.00
Structure contains 15 cavities	Structure contains 2 cavities

MD simulation was applied to confirm the reliability of each ligand into the active site of the enzymes. The fresh identified hit compounds formed stable hydrogen bond interactions with the modeled active residues, e.g., Glu299 and Met6 for SARS-CoV-2 main protease (Fig. 10D) and Tyr19 for RNA polymerase-binding transcription factor DksA (Fig. 11D). The MD simulation also supported that each hit compound formed hydrophobic interactions with residues occupying the active site of SARS-CoV-2 main protease and RNA polymerase-binding transcription factor. Eventually, we proposed two-hit compounds as key practical weapons for the COVID-19 main protease and RNA polymerase therapeutics against SARS-CoV-2 and *Pseudomonas aeruginosa* inhibition, respectively.

## Conclusion

The created 2D-QSAR models’ regression statistics demonstrated that they were statistically significant. Furthermore, during fitting’s criteria, internal, and external cross-validation trials, relatively low residuals were acquired, showing that the constructed models were predictive. Their satisfactory QL2OO, R2, Q2F1, Q2F2, Q2F3, and CCC values backed up this claim. In docking simulation, compounds 15 and 18 were predicted as the best RNA polymerase-binding transcription factor and SARS-CoV-2 virus main protease inhibitor, respectively (with maximum binding affinity) to be employed as a possible cure orally active drug (based on BOILED-egg and bioavailability radar approach). Molecular dynamic simulations analyze admitting RMSD, RMSF, and SASA analysis affirmed their binding constancy with respective modeled proteins throughout the simulation chronology. Our present exploit can be generative in determining new remedies against SARS-CoV-2 virus main protease and *Pseudomonas aeruginosa*, having said that general test (in vitro and in vivo) studies are required to test our theoretical analysis.

## Supplementary Information

**Additional file 1: Table S1.** Chemical structures of iminoguanidine compounds as well as their activity levels. **Table S2.** The regression statistics of the 2D-QSAR equations. **Table S3.** Experimental endpoint and predicted pMIC50 values of training and test set compounds by Model 1 equation. **Table S4.** The binding affinity score of each ligand and standards in SARS-CoV-2 virus and pseudomonas aeruginosa using the Autodock vina with PyRx program.

**Additional file 2: Figure S1.** The plot of experimental endpoint *vs* predicted pMIC_50_ by model equation. **Figure S2.** The plot of experimental endpoint *vs* predicted pMIC_50_ LOO. **Figure S3.** View residuals calculated using predictions by model equation. **Figure S4.** View Residuals calculated using predictions by LOO. **Figure S5.** Using h* = 0.5 as the warning leverage, the plot of standardized residuals versus hat values (William plot). **Figure S6.** Using h* = 0.5 as the warning leverage, the plot of standardized residuals versus hat values (William plot – Prediction by LOO). **Figure S7.** Insubria Graph for the applicability domain inspection of the developed model. **Figure S8.** Leave-two-out cross validation *vs* Kxy. **Figure S9.** Plot of Y-scrambled validations models compared with the original model. **Figure S10.** Y-randomization validation procedure to verify chance correlation of a model using QSAR modeling. **Figure S11.** Quality verification plot of the energy minimized model of the19-fatty acid desaturase performed using ERRAT.

## Data Availability

Not applicable

## Abbreviations

QSAR: Quantitative structure-activity relationship
GFA: Genetic function approximation
MLR: Multiple linear regression
MDS: Molecular dynamics simulation
ADMET: Absorption, Distribution, Mechanism, Excretion, and Toxicity
PDB: Protein Data Bank
RMSD: Root mean square deviation
RMSF: Root mean square fluctuation
SASA: Solvent accessible surface area
