# Supplementary material for: QSAR, homology modeling, and docking simulation on SARS-CoV-2 and pseudomonas aeruginosa inhibitors, ADMET, and molecular dynamic simulations to find a possible oral lead candidate
Source: J Genet Eng Biotechnol. 2022 Jun 17;20:88. doi: 10.1186/s43141-022-00362-z (PMC9205150; doi:10.1186/s43141-022-00362-z)
Supplement: Supplementary file 1 — Additional file 1: Table S1. Chemical structures of iminoguanidine compounds as well as their activity levels. Table S2. The regression statistics of the 2D-QSAR equations. Table S3. Experimental endpoint and predicted pMIC50 values of training and test set compounds by Model 1 equation. Table S4. The binding affinity score of each ligand and standards in SARS-CoV-2 virus and pseudomonas aeruginosa using the Autodock vina with PyRx program. [file 43141_2022_362_MOESM1_ESM.docx]

Table S1. Chemical structures of iminoguanidine compounds as well as their activity levels.

| Compound Number | Structures | MIC50 | Activity |
| --- | --- | --- | --- |
|  | Accession Number AID_131512 | ug/mL | pMIC_50_ |
| 1 |  | 49 | 1.69019608 |
| 2 |  | 121 | 2.08278537 |
| 3 |  | 123 | 2.089905111 |
| 4 |  | 99.5 | 1.997823081 |
| 5 |  | 72.8 | 1.862131379 |
| 6 |  | 131.2 | 2.117933835 |
| 7 |  | 48.4 | 1.684845362 |
| 8 |  | 52.4 | 1.719331287 |
| 9 |  | 47.3 | 1.674861141 |
| 10 |  | 52.3 | 1.718501689 |
| 11 |  | 113 | 2.053078443 |
| 12 |  | 32.1 | 1.506505032 |
| 13 |  | 162 | 2.209515015 |
| 14 |  | 52.5 | 1.720159303 |
| 15 |  | 23.2 | 1.365487985 |
| 16 |  | 50.1 | 1.699837726 |
| 17 |  | 80.5 | 1.90579588 |
| 18 |  | 59.1 | 1.771587481 |
| 19 |  | 1140 | 3.056904851 |
| 20 |  | 408 | 2.610660163 |
| 21 |  | 750 | 2.875061263 |
| 22 |  | 134 | 2.127104798 |
| 23 |  | 66.6 | 1.823474229 |
| 24 |  | 51.3 | 1.710117365 |
| 25 |  | 32.3 | 1.509202522 |
| Azithromycin |  |  |  |
| Doxycycline |  |  |  |
| Levofloxacin |  |  |  |
| Fluoroquinolone |  |  |  |
| Chloroquine |  |  |  |
| Ritonavir |  |  |  |
| Ruxolitnib |  |  |  |
| Ampicillin |  |  |  |

Table S2. The regression statistics of the 2D-QSAR equations.

| Validation criteria | Model scores | Threshold | Remark |
| --- | --- | --- | --- |
| Fitting’s criteria |  |  |  |
| R2tr | 0.8735 | R2tr ≥ 0.6 | Pass |
| R2adj | 0.8566 | R2adj ≥ 0.5 | Pass |
| R2-R2adj | 0.0169 | R2tr–R2adj ˂ 0.1 | Pass |
| LOF | 0.0329 | Low | Pass |
| Kxx | 0.3095 | Low | Pass |
| Delta K | 0.3060 | Low | Pass |
| RMSE tr | 0.1411 | RMSE tr ˂ RMSE cv | Pass |
| MAE tr | 0.0966 | close to zero | Pass |
| RSS tr | 0.3582 |  | Pass |
| CCC tr | 0.9325 | CCC tr ≥ 0.8 | Pass |
| s | 0.1545 | Low | Pass |
| F | 51.7716 | Large | Pass |
| Internal validation criteria | |  |  |
| Q2loo | 0.8027 | Q2LOO ≥ 0.5 | Pass |
| R2-Q2loo | 0.0707 | R2 – Q2LOO ≤ 0.1 | Pass |
| RMSE cv | 0.1761 | Close to zero | Pass |
| MAE cv | 0.1204 | Close to zero | Pass |
| PRESS cv | 0.5585 |  | Pass |
| CCC cv | 0.8937 | CCC cv ≥ 0.8 | Pass |
| Q2LMO | 0.7658 | Q2LMO ≥ 0.6 | Pass |
| R2Yscr | 0.1182 | R2Yscr ˂ R2 tr | Pass |
| Q2Yscr | -0.3102 | Q2Yscr ˂ Q2LOO | Pass |
| RMSE AV Yscr | 0.3715 |  | Pass |
| R2Yrnd | 0.1166 | R2Yscr ˂ R2tr | Pass |
| Q2Yrnd | -0.3187 | Q2Yscr ˂ Q2LOO | Pass |
| External validation criteria | |  |  |
| RMSE ext | 0.4657 | Close to zero | Pass |
| MAE ext | 0.3835 | Close to zero | Pass |
| PRESS ext | 1.3010 |  | Pass |
| R2ext | 0.7536 | R2ext ≥ 0.6 | Pass |
| Q2-F1 | -0.0110 |  | Pass |
| Q2-F2 | -0.7217 |  | Pass |
| Q2-F3 | -0.3786 |  | Pass |
| CCC ext | 0.6769 | CCC ext ≥ 0.8 | Pass |
| r2m aver. | 0.2029 |  | Pass |
| r2m delta | 0.5434 |  | Pass |
| Predictions by LOO:  Exp(x) vs. Pred(y): R2 = 0.8036; R'2o = 0.7717; k' = 0.9898; Clos' = 0.0397; r'2m = 0.6600  Pred(x) vs. Exp(y): R2 = 0.8036; R2o = 0.8028; k = 0.9990; Clos = 0.0010; r2m = 0.7807 | | | |
| External predictions by the model equation:  Exp(x) vs. Pred(y): R2 = 0.7536; R'2o = 0.6165; k' = 1.0471; Clos' = 0.1819; r'2m = 0.4746  Pred(x) vs. Exp(y): R2 = 0.7536; R2o = -0.4373; k = 0.9094; Clos = 1.5803; r2m = -0.0688 | | | |

Table S3. Experimental endpoint and predicted pMIC50 values of training and test set compounds by Model 1 equation.

| ID | Name | Status | Exp. endpoint | Pred. by model eq. | Pred. Mod. Eq. Res. | Pred. LOO | Pred. LOO Res. | HAT i/i (h*=0.5000) | Std. Pred. Mod. Eq. Res. | Std. Pred. LOO Res. |
| --- | --- | --- | --- | --- | --- | --- | --- | --- | --- | --- |
| 1 | 2 | Prediction | 1.5119 | 1.4139 | -0.0980 | - | - | 0.1023 | -0.6693 | -0.6693 |
| 2 | 3 | Training | 1.7672 | 1.8992 | 0.1320 | 2.0274 | 0.2602 | 0.4926 | 1.1996 | 2.3642 |
| 3 | 4 | Training | 1.5843 | 1.5695 | -0.0148 | 1.5685 | -0.0158 | 0.0608 | -0.0991 | -0.1056 |
| 4 | 5 | Training | 1.7882 | 1.7127 | -0.0755 | 1.6951 | -0.0930 | 0.1886 | -0.5423 | -0.6683 |
| 5 | 6 | Training | 1.4564 | 1.3419 | -0.1145 | 1.3271 | -0.1293 | 0.1146 | -0.7873 | -0.8892 |
| 6 | 7 | Training | 1.5011 | 1.5624 | 0.0614 | 1.5792 | 0.0782 | 0.2149 | 0.4483 | 0.5710 |
| 7 | 8 | Training | 1.2833 | 1.2865 | 0.0032 | 1.2869 | 0.0036 | 0.1081 | 0.0220 | 0.0247 |
| 8 | 9 | Training | 1.2122 | 1.2389 | 0.0268 | 1.2426 | 0.0304 | 0.1212 | 0.1847 | 0.2102 |
| 9 | 10 | Training | 1.3139 | 1.3214 | 0.0075 | 1.3223 | 0.0084 | 0.1031 | 0.0515 | 0.0574 |
| 10 | 11 | Training | 1.4843 | 1.8071 | 0.3228 | 1.8393 | 0.3550 | 0.0908 | 2.1903 | 2.4091 |
| 11 | 12 | Training | 1.4829 | 1.3718 | -0.1110 | 1.3568 | -0.1261 | 0.1193 | -0.7656 | -0.8693 |
| 12 | 13 | Prediction | 1.9415 | 2.3945 | 0.4530 | - | - | 1.1057 |  |  |
| 13 | 14 | Training | 1.2945 | 1.2389 | -0.0556 | 1.2311 | -0.0634 | 0.1231 | -0.3840 | -0.4379 |
| 14 | 15 | Training | 1.4065 | 1.6956 | 0.2891 | 1.7179 | 0.3114 | 0.0716 | 1.9415 | 2.0912 |
| 15 | 16 | Prediction | 1.7987 | 1.2057 | -0.5929 | - | - | 0.1285 | -4.1101 | -4.1101 |
| 16 | 17 | Prediction | 2.1614 | 1.9309 | -0.2305 | - | - | 0.5687 | -2.2710 | -2.2710 |
| 17 | 18 | Prediction | 1.5391 | 1.4311 | -0.1080 | - | - | 0.1280 | -0.7481 | -0.7481 |
| 18 | 19 | Prediction | 2.5289 | 3.3473 | 0.8184 | - | - | 1.4583 |  |  |
| 19 | 20 | Training | 2.6464 | 2.6323 | -0.0141 | 2.6188 | -0.0276 | 0.4898 | -0.1278 | -0.2505 |
| 20 | 21 | Training | 2.5922 | 2.2824 | -0.3098 | 2.1549 | -0.4373 | 0.2916 | -2.3816 | -3.3619 |
| 21 | 22 | Training | 1.6866 | 1.7131 | 0.0264 | 1.7149 | 0.0282 | 0.0633 | 0.1768 | 0.1887 |
| 22 | 23 | Training | 1.8451 | 1.8009 | -0.0442 | 1.7936 | -0.0515 | 0.1413 | -0.3085 | -0.3593 |
| 23 | 24 | Training | 1.3617 | 1.2384 | -0.1233 | 1.2217 | -0.1401 | 0.1197 | -0.8504 | -0.9660 |
| 24 | 25 | Training | 1.3820 | 1.3756 | -0.0064 | 1.3750 | -0.0070 | 0.0856 | -0.0435 | -0.0476 |

Table S4. The binding affinity score of each ligand and standards in SARS-CoV-2 virus and pseudomonas aeruginosa using the Autodock vina with PyRx program

| Compound No. | SARS-CoV-2 virus | pseudomonas aeruginosa |
| --- | --- | --- |
| Protein-Ligand Complex | Binding Affinity (kcal/mol) | Binding Affinity  (kcal/mol) |
| predprotein_1 | -6.8 | -5.2 |
| predprotein_2 | -7.7 | -5.5 |
| predprotein_3 | -7.1 | -5.9 |
| predprotein_4 | -6.9 | -5.8 |
| predprotein_5 | -7.1 | -5.6 |
| predprotein_6 | -7.1 | -6 |
| predprotein_7 | -8.4 | -6.5 |
| predprotein_8 | -6.9 | -5.4 |
| predprotein_9 | -6.5 | -5.9 |
| predprotein_10 | -7.9 | -6 |
| predprotein_11 | -7.4 | -5.3 |
| predprotein_12 | -8.1 | -6.5 |
| predprotein_13 | -7.3 | -6 |
| predprotein_14 | -6.8 | -5.2 |
| predprotein_15 | -8.5 | -6.5 |
| predprotein_16 | -6.5 | -5.9 |
| predprotein_17 | -7 | -5.4 |
| predprotein_18 | -8.7 | -6.3 |
| predprotein_19 | -7 | -5.6 |
| predprotein_20 | -7.2 | -5.3 |
| predprotein_21 | -7.3 | -5.7 |
| predprotein_22 | -7.1 | -5.5 |
| predprotein_23 | -7.1 | -5.7 |
| predprotein_24 | -7 | -5.6 |
| predprotein_25 | -6.9 | -5.2 |
| Azithromycin | -7.7 | -6.1 |
| Doxycycline | -7.5 | -7.2 |
| Levofloxacin | -7.8 | -6.3 |
| Fluoroquinolone | -6.1 | -5 |
| Chloroquine | -6.7 | -4.6 |
| Ritonavir | -8.4 | -6.7 |
| Ruxolitnib | -6.8 | -5.9 |
| Ampicillin | -6.7 | -6.1 |
