# Supplementary material for: QSAR, homology modeling, and docking simulation on SARS-CoV-2 and pseudomonas aeruginosa inhibitors, ADMET, and molecular dynamic simulations to find a possible oral lead candidate
Source: J Genet Eng Biotechnol. 2022 Jun 17;20:88. doi: 10.1186/s43141-022-00362-z (PMC9205150; doi:10.1186/s43141-022-00362-z)
Supplement: Supplementary file 2 — Additional file 2: Figure S1. The plot of experimental endpoint vs predicted pMIC50 by model equation. Figure S2. The plot of experimental endpoint vs predicted pMIC50 LOO. Figure S3. View residuals calculated using predictions by model equation. Figure S4. View Residuals calculated using predictions by LOO. Figure S5. Using h* = 0.5 as the warning leverage, the plot of standardized residuals versus hat values (William plot). Figure S6. Using h* = 0.5 as the warning leverage, the plot of standardized residuals versus hat values (William plot – Prediction by LOO). Figure S7. Insubria Graph for the applicability domain inspection of the developed model. Figure S8. Leave-two-out cross validation vs Kxy. Figure S9. Plot of Y-scrambled validations models compared with the original model. Figure S10. Y-randomization validation procedure to verify chance correlation of a model using QSAR modeling. Figure S11. Quality verification plot of the energy minimized model of the19-fatty acid desaturase performed using ERRAT. [file 43141_2022_362_MOESM2_ESM.docx]

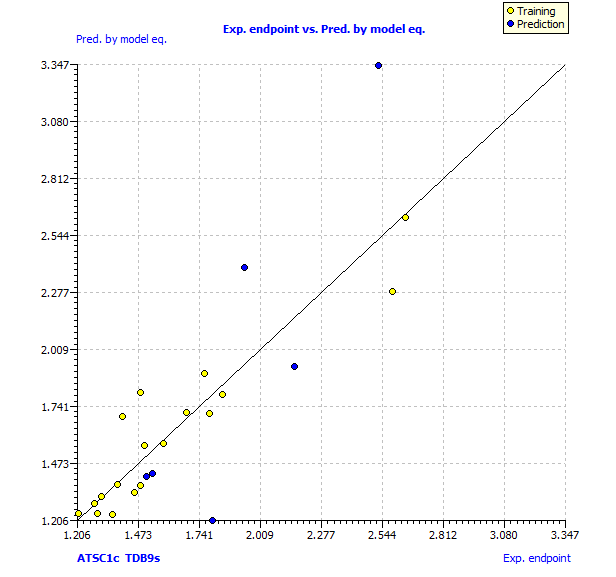


Fig. S1. The plot of experimental endpoint *vs* predicted pMIC_50_ by model equation


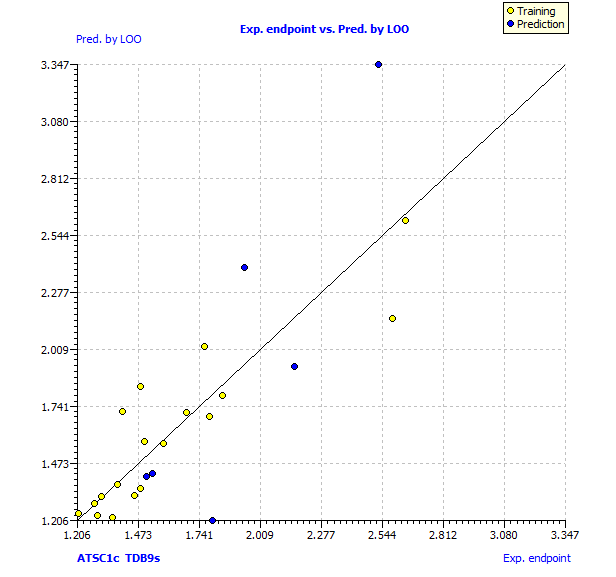


Fig. S2. The plot of experimental endpoint *vs* predicted pMIC_50_ LOO


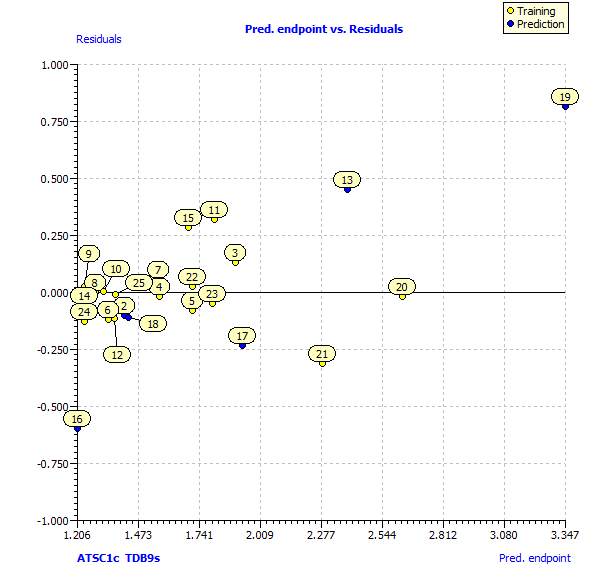


Fig. S3. View residuals calculated using predictions by model equation


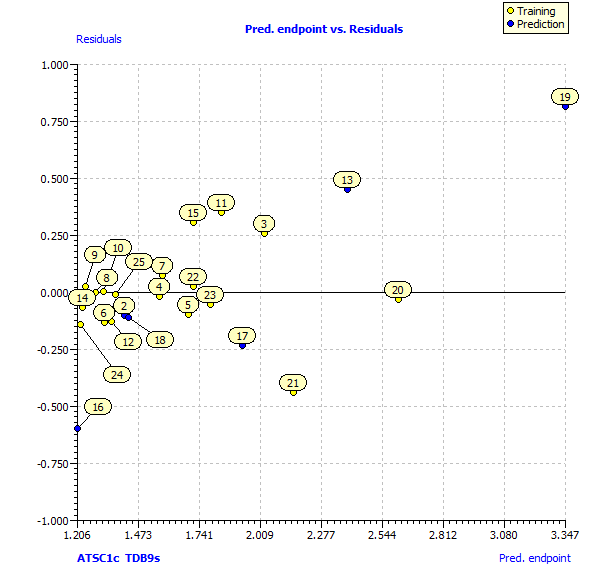


Fig. S4. View Residuals calculated using predictions by LOO


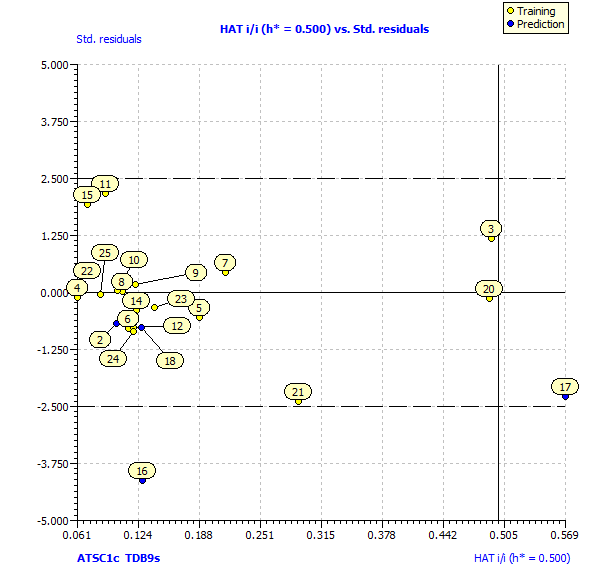


Fig. S5. Using h* = 0.5 as the warning leverage, the plot of standardized residuals versus hat values (William plot).


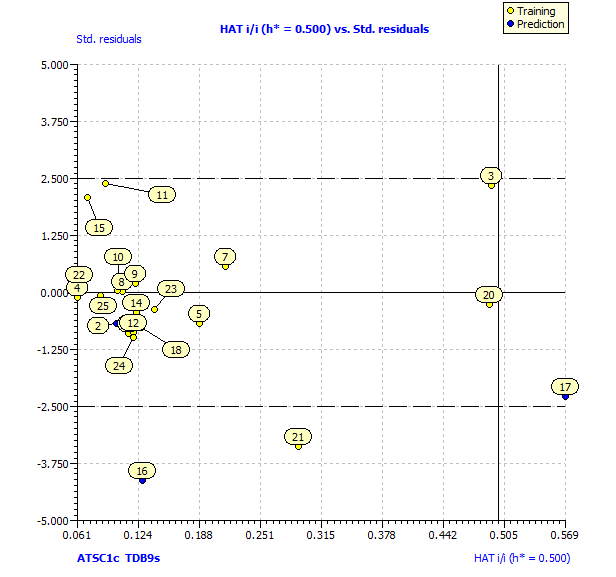


Fig. S6. Using h* = 0.5 as the warning leverage, the plot of standardized residuals versus hat values (William plot – Prediction by LOO).


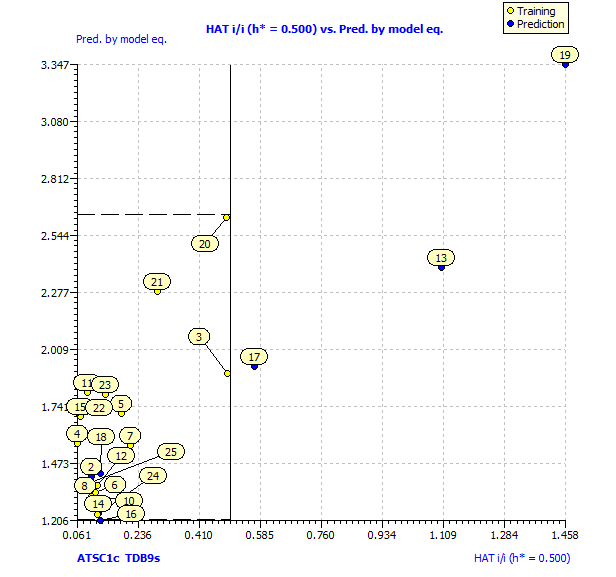


Fig. S7. Insubria Graph for the applicability domain inspection of the developed model


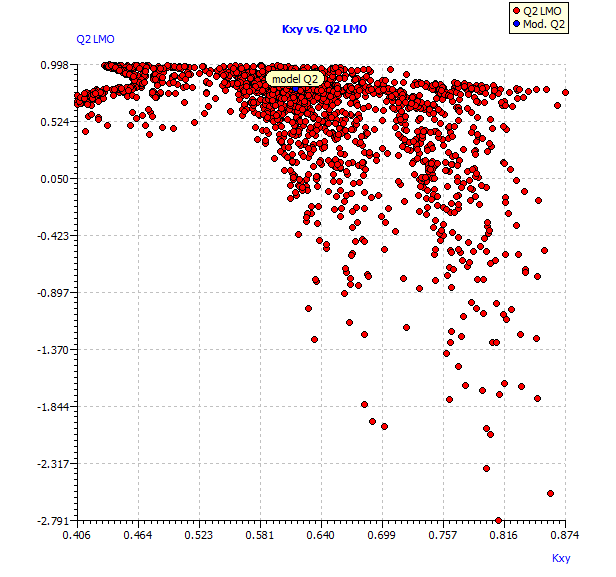


Fig. S8. Leave-two-out cross validation *vs* Kxy


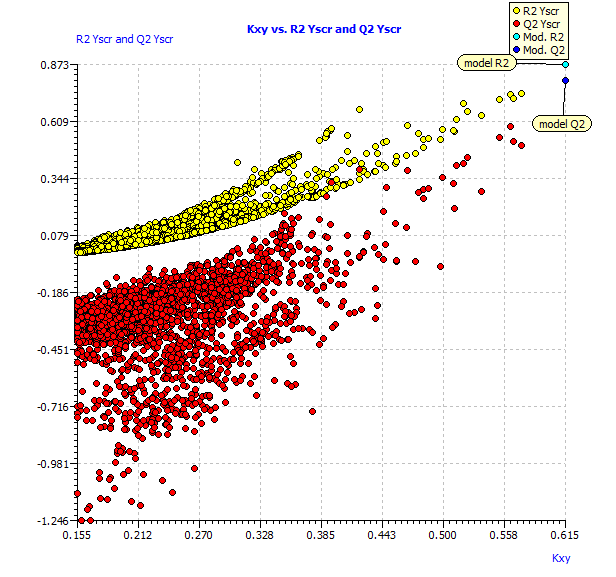


Fig. S9. Plot of Y-scrambled validations models compared with the original model.


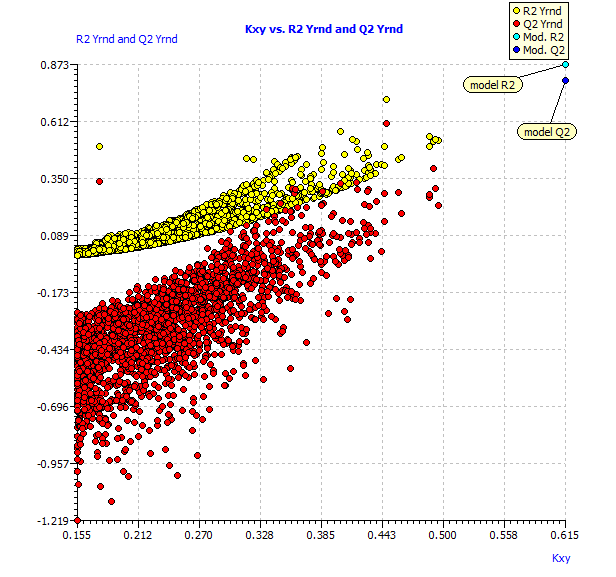


Figure S10. Y-randomization validation procedure to verify chance correlation of a model using QSAR modeling.

| 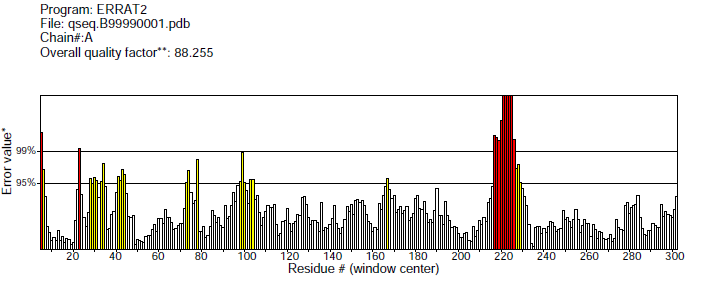A |
| --- |
| 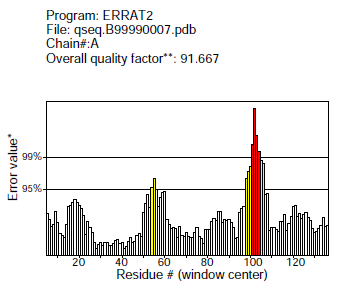**B** |

Fig. S11. Quality verification plot of the energy minimized model of the19-fatty acid desaturase performed using ERRAT.
